# Anatomical Variations in Root Canal Configuration of Maxillary Second Premolars: A Narrative Review

**DOI:** 10.3390/dj14060369

**Published:** 2026-06-15

**Authors:** Michał Głąbski, Monika Kuczmaja, Agata Żółtowska

**Affiliations:** Department of Conservative Dentistry, Faculty of Medicine, Medical University of Gdańsk, 80-210 Gdańsk, Poland; michal.glabski@gumed.edu.pl

**Keywords:** canal configuration, second premolars, CBCT

## Abstract

The maxillary second premolar, while frequently single rooted, exhibits a high degree of morphological diversity in its internal canal system. This unpredictability can lead to clinical oversights if a simple anatomy is assumed. The aim of this study was to review current knowledge regarding the number of roots and root canal configurations in permanent maxillary second premolars across different populations. A comprehensive literature search was conducted using the PubMed database for studies published between 2015 and December 2025, with the keyword “maxillary second premolar anatomy.” Out of 358 identified articles, 27 studies met the inclusion criteria and were analyzed. Only human-based studies on maxillary second premolars that used CBCT imaging and were written in English were included. The reviewed studies revealed that single-rooted maxillary second premolars are the most prevalent morphology, occurring in over 70% of cases, although significant variations exist among different ethnic groups. The presence of two roots was the second most common configuration, while three-rooted teeth were rare (<2%). Gender-related differences were also observed, with a higher prevalence of two-rooted teeth in males. Analysis of root canal configurations based on Vertucci’s classification demonstrated that all eight types can occur, with Type I being the most frequent in most populations with a result of more than 50% of all teeth. However, substantial variability was noted, with certain studies reporting a higher prevalence of more complex configurations such as Types IV and V. The findings emphasize the importance of thorough knowledge of root canal anatomy and its variations to ensure successful endodontic outcomes. Advanced imaging techniques, particularly cone beam computed tomography (CBCT), play a crucial role in improving diagnostic accuracy and proper endodontic treatment. Further research is needed to better understand anatomical differences across populations and enhance clinical decision-making in endodontics.

## 1. Introduction

The loss of a permanent tooth can lead to disruption of this system, as well as problems with articulation and facial appearance [1].

Modern dentistry strives to retain as many teeth as possible. Removing a permanent tooth leads to disruption of the stomatognathic system.

The stomatognathic system is a morphological and functional complex of interacting anatomical structures of the oral cavity and facial skeleton [2].

The key to successful treatment is proper treatment of all root canals. Adequate treatment enables effective bacterial eradication and proper obturation of the root canal system, thereby improving treatment outcomes. Knowledge of the anatomy, configuration of the root canal system and the ability to find the orifices of all canals is crucial for a dentist. Anatomical changes in the form of additional canals [3], isthmuses [4], and curvatures [5] can pose significant challenges in thorough cleaning and preparation of canals. Therefore, knowledge of the number and distribution of root canal configurations in a given population can help the dentist to perform the appropriate treatment. Although maxillary second premolars are frequently perceived as anatomically simple teeth, numerous studies have demonstrated considerable variability in root number and canal configuration. Assuming a single-rooted tooth with one root canal may increase the risk of missed anatomy and endodontic failure. However, studies show that in specific populations, complex configurations such as Vertucci type IV or V can occur in nearly 30% of cases. Some methods of saving a tooth in the oral cavity include conservative treatment, which involves treating caries and filling and root canal treatment.

Failure to locate all root canals during treatment may lead to persistent infection and pain [6,7,8]. Residual bacteria within untreated canals [9] may continue to proliferate, contributing to the development of periapical lesions and potentially necessitating retreatment. In addition, failure to identify a canal may result in its gradual obliteration, which can significantly complicate future treatment and may require more advanced approaches, such as guided endodontic therapy [10]. Furthermore, the application of platelet-rich fibrin (PRF) has gained increasing attention in endodontics due to its regenerative properties and its potential to enhance periapical healing and treatment outcomes [11]. Surgical endodontic intervention may also be indicated [6], and, in advanced cases, tooth extraction may become unavoidable. Such outcomes are associated with compromised esthetics and may adversely affect the patient’s overall quality of life and functional comfort. From a clinical perspective, untreated canals may compromise disinfection and obturation, thereby increasing the risk of persistent apical periodontitis, retreatment, and reduced long-term treatment success. Therefore, understanding anatomical variability is of particular importance in endodontic diagnosis and treatment planning.

The European Society of Endodontics recommends the use of X-rays for endodontic treatment, as they are readily available and widely used in clinics.

Their main drawback is that they only provide two-dimensional imaging. Such imaging can produce many false positives by obscuring superimposed images and structures. Therefore, cone beam computed tomography (CBCT) is an increasingly popular method for imaging the configuration of tooth canals and roots. CBCT uses a conical beam of radiation, which significantly reduces the radiation dose compared to a traditional CT scanner [12].

In traditional computed tomography, a slit beam of X-rays cuts slice by slice from the examined object, repeatedly circling the patient’s head in a spiral path. This examination utilizes cone beam computed tomography (CBCT), which allows for the three-dimensional imaging of tooth tissues and surrounding structures. In the DVT technique, the radiation beam has the shape of a cone and illuminates not the surface, but the examined volume of the patient, during a single rotation around his head. This imaging demonstrates the diversity of canal system configurations and allows for the assessment of accessory canals and root deltas.

CBCT also demonstrates substantially higher sensitivity in detecting inflammation of apical periodontitis [13].

A small imaging field of view (FOV) with a small voxel size such as 5 cm × 5 cm is recommended as the best acquisition parameter in endodontics. This allows for increased sensitivity in detecting pathology while limiting the radiation dose in accordance with the ALARA principle [14].

The ALARA and ALADA principles are crucial in radiological examination [15].

Knowledge about the number, configuration and frequency of occurrence of individual canal configurations is crucial in endodontic treatment. Scientific research on the configuration of root canals has been conducted since the 1960s.

Root canal morphology has been described using several classification systems, most notably those proposed by Weine et al. [16], Vertucci [17], and Ahmed et al. [18]. The classification developed by Weine et al. [16] represented one of the first systematic approaches to categorizing root canal configurations. Initially, three canal patterns were described in 1969 [16], and a fourth configuration was later incorporated into the system in 1982 [19].

**Figure 1:**
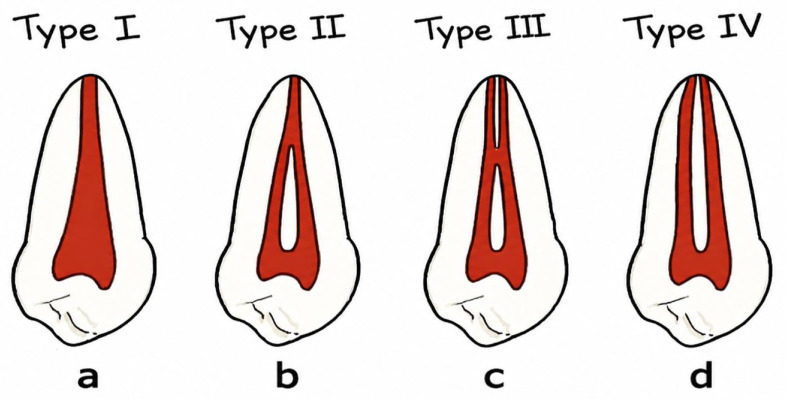
Wein configuration 1984.

Wein’s classification distinguishes four types. In Type I (Figure 1a), the canal begins with an orifice in the pulp chamber, runs along the entire length of the root and ends with one anatomical opening. In Type II (Figure 1b), two canals begin with separate openings in the pulp chamber, run through the root, connect and end with one common foramen in the root apex. In Type III (Figure 1c), two canals have a separate opening in the pulp chamber, run separately through the root and end with their own anatomical foramen. Type IV (Figure 1d) is characterized by one opening in the pulp chamber, the canal running through the root is divided into two canals that end in two separate anatomical foramina.

**Figure 2:**
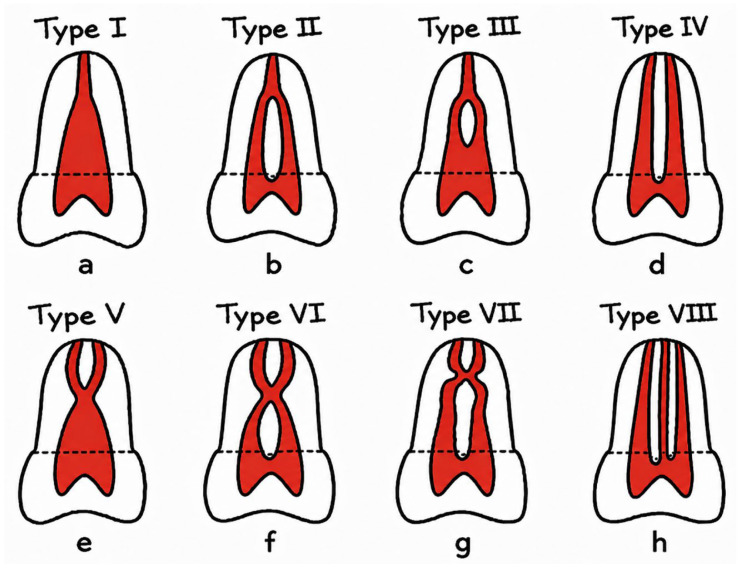
Vertucci configuration.

Vertucci’s classification of canals, which distinguishes eight types of root canal systems, has been since late 1980s (Figure 2).

In Type I (Figure 2a), the canal begins with an orifice in the pulp chamber, runs along the entire length of the root and ends with one anatomical opening. In Type II (Figure 2b) two canals begin with separate openings in the pulp chamber, run through the root, connect and end with one common foramen in the root apex. In Type III (Figure 2c), there is one canal opening in the pulp chamber; as it runs through the root, the canal divides into two canals, which then merge into one again and end with one foramen in the root apex. In Type IV (Figure 2d) two canals have a separate opening in the pulp chamber, run separately through the root and end with their own anatomical foramen.

Type V (Figure 2e) is characterized by one opening in the pulp chamber, the canal running through the root is divided into two canals that end in two separate anatomical foramina.

In Type VI (Figure 2f), two canals begin with individual orifices in the pulp chamber, merge along their course into one canal, then separate again and end with two separate foramina in the root apex.

In Type VII (Figure 2g), there is one opening in the pulp chamber, then the canal divides into two, then merges into one and divides again into two canals ending with separate foramina in the root apex.

Type VIII (Figure 2h) is characterized by the presence of three separate orifices in the pulp chamber, an individual course of three canals that end in separate foramina in the root apex.

Since its introduction in 2017, Ahmed’s classification [18], which more precisely defines the number of canals, their connecting roots, and their openings, has gained importance, particularly in Middle Eastern countries. Ahmed’s classification uses a code containing the tooth number, number of roots and the exact configuration of root canals with their connections and divisions. The system describes the canal from the opening of the root canal (Orifice), through the canal (Canal), to the apical foramen (Foramen)-[TN^O−C−F^]. The number of roots is added as a superscript before the tooth number [^R^TN]. Root canal configurations in each root if more than one root are added after the tooth number.

An incorrect assumption regarding the number of canals can lead to treatment failure due to incomplete eradication of bacteria from the root canal system and leaky filling, which results in treatment failure [20].

Assuming the presence of a single root canal in maxillary second premolars may be misleading, as confirmed by numerous studies conducted by many authors, which also demonstrate the existence of two- and three-rooted teeth. The anatomy of these teeth is variable, which affects the predictability of endodontic treatment; the presence of one root canal cannot be assumed in each case, so a review is necessary in order to demonstrate the presence of other canal and consequently to plan endodontic treatment more precisely.

The aim of this narrative review was to summarize current evidence regarding the anatomical variability of permanent maxillary second premolars, with particular emphasis on two investigated units: (1) the number of roots and (2) the number and configuration of root canals based on cone beam computed tomography (CBCT) studies. This review aimed to assess the prevalence of root number and root canal configurations among different populations and ethnic groups, as well as sex-related differences in anatomical morphology.

The null hypothesis assumed that there is no association between sex and (1) the number of roots and (2) root canal configuration in maxillary second premolars.

## 2. Materials and Methods

**Figure 3:**
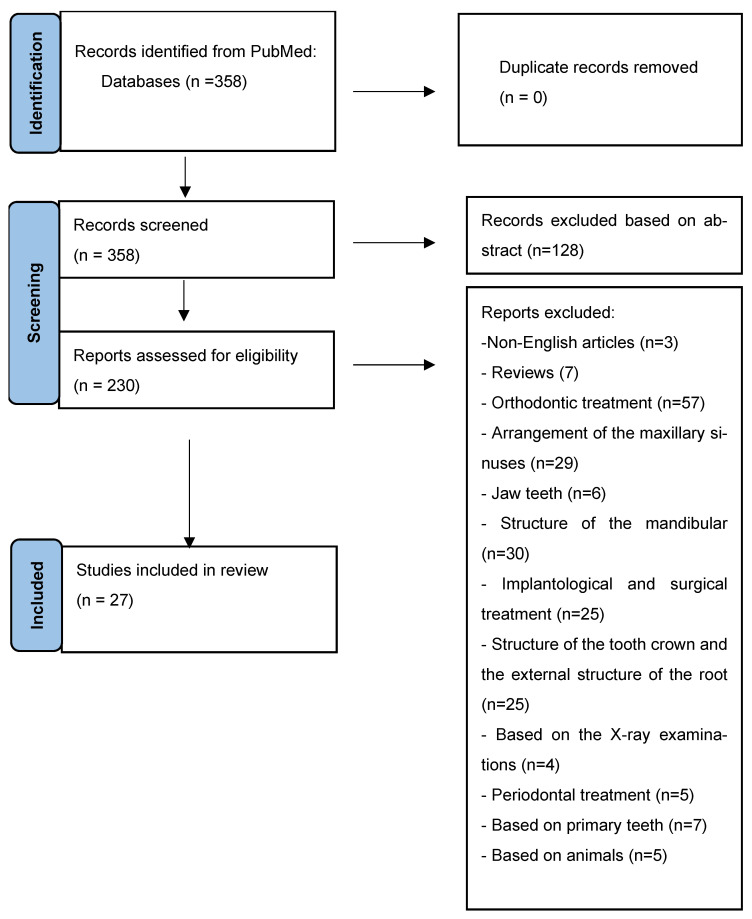
PRISMA flow chart of the selection process.

This article was designed as a narrative review aimed at providing a clinically oriented synthesis of currently available evidence regarding anatomical variations in maxillary second premolars. A narrative approach was selected due to heterogeneity among studies with respect to populations, CBCT protocols, sample size, and classification systems. The literature search was primarily conducted using PubMed as the principal database for biomedical research between 2015 and December 2025, with the keyword ‘maxillary second premolar anatomy. This specific decade was chosen to focus on data obtained through high-resolution cone beam computed tomography (CBCT). Modern CBCT imaging with small Field of View (FOV) and reduced voxel size provides significantly more accurate visualization of root canal systems than the methodologies used in earlier eras. Out of 358 identified articles, no duplicates were obtained. A total of 358 records were screened, having considered its abstract 128 were excluded. A total of 230 records were assessed for eligibility; 203 of them were excluded, and, of these, 128 were off topic, 7 of them concerned reviews, 3 were in a language other than English, 57 concerned orthodontic treatment, 29 concerned the arrangement of the maxillary sinuses, 6 concerned the jaw teeth, 30 examined the structure of the jaw bone, 25 involved implantological and surgical treatment, 25 concerned the structure of the tooth crown and the external structure of the root, 4 were conducted on the basis of X-ray examinations, 5 concerned treatment periodontology, 7 concerned primary teeth in children, and 5 concerned studies conducted on animals (Figure 3). A total of 27 studies met the inclusion criteria and were included in the qualitative synthesis. However, only 21 of these provided specific numerical data regarding root and canal morphology suitable for quantitative comparison. Consequently, the remaining 6 studies were utilized for descriptive analysis of anatomical variations and clinical recommendations, but are not listed in the comparative tables (Table 1 and Table 2). Only human-based studies involving maxillary second premolars that were written in English were included. The primary inclusion criterion for the selected studies was the use of CBCT as the diagnostic tool for assessing root canal morphology. CBCT was prioritized due to its superior accuracy in identifying complex canal configurations compared to traditional two-dimensional radiography.

Due to substantial heterogeneity among included studies regarding methodology, imaging protocols, and reported outcomes, a formal meta-analysis was not performed. Instead, the findings were narratively synthesized with emphasis on clinically relevant anatomical patterns.

## 3. Discussion

### 3.1. Sample Size

The included studies varied significantly in terms of sample size, with the number of examined maxillary second premolars (Table 1). The heterogeneity test revealed a high level of inconsistency among the results (I^2^ = 97.9%), indicating substantial variability between the studies. The substantial heterogenity observed among the studies may be explained by methodological differences, including variations in sample size. In addition, ethnic diversity among the analyzed populations likely contributed to differences in the prevalence of the number of roots in second maxillary premolars.

The included studies demonstrated substantial variability in sample size. Among the investigations evaluating root number, the largest cohorts were reported by Li YH et al. [42], Algarni et al. [37], and Watanabe et al. [27], whereas smaller sample sizes were observed in the studies conducted by Estrela et al. [39], Buchanan et al. [29], and Mustafa et al. [35]. A similar pattern was noted in studies assessing root canal configuration, with the largest cohorts reported by Li YH et al. [42], Ok E et al. [45], and Watanabe et al. [27], while the smallest sample sizes were observed in the studies by Estrela et al. [39], Lemos et al. [44], and Buchanan et al. [29]. Age-related analyses were performed by Algarni et al. [37], Mustafa et al. [35], and Ok E et al. [45]. None of these studies demonstrated a significant association between age and either root number or root canal configuration. Overall, the reviewed investigations confirmed considerable anatomical variability in maxillary second premolars, encompassing a broad range of canal configurations according to the Vertucci classification. In addition, rare anatomical variants have been documented, including a case of a maxillary second premolar with four separate root canals reported by Izaz et al. [46].

### 3.2. Root Number Distribution Across Different Populations

Based on the available studies, it can be concluded that the research was conducted across different continents and among diverse ethnic groups. The included studies involved diverse ethnic populations across multiple continents. In some regions, such as Saudi Arabia, several studies were conducted, with varying results over several years.

The Saudi Arabian subpopulation showed the highest single-root stability of 99.7% in the study conducted by Mustafa et al. [35]. It is worth noting that several studies were conducted in Saudi Arabia by Elnour et al. [43] Chourasia et al. [26], Mashyakhy et al. [32], and Mustafa et al. [35] (Table 1). Considerable variability was observed among the reported findings. According to Chourasia et al. [26] (Table 1), the prevalence of two-rooted maxillary second premolars was 20.76%, according to Mashyakhy et al. [32] (Table 1), this was 12%, and in Mustafa et al.’s [35] (Table 1) study, this was 0.3%. The authors did not consider gender.

The results collected in Japan by Watanabe et al. [27] and in South Korea by Jung et al. [28] did not differ at all. In these regions, single-rooted second premolars had a prevalence of 97% (Table 1), and the prevalence of two roots was higher in men than in women (Table 3).

Across all included studies, the presence of a single root is reported in over 70%, while the second most common root configuration is the presence of two roots, and the three-root configuration occurs in less than 2% of cases. Only Almehrzi et al.’s [36] study demonstrated the presence of a C-SHAPE root (0.6%) (Table 1).

The observed differences among populations may reflect ethnic and genetic diversity, which has previously been associated with variation in root morphology. However, methodological heterogeneity should also be considered when interpreting these findings. Differences in CBCT acquisition parameters, voxel size, sample composition, and inclusion criteria may partially explain discrepancies between studies. Furthermore, the considerable variation observed even within the Saudi Arabian population suggests that anatomical prevalence may not be generalized across geographically related populations and may instead reflect regional subpopulations or methodological variability. Overall, the findings indicate that although a single-rooted morphology predominates worldwide, substantial inter-population differences exist. Populations from East Asia generally demonstrated a higher prevalence of single-rooted teeth, whereas studies conducted in the Middle East, South America, and parts of Africa reported greater anatomical diversity. These observations suggest that both genetic background and ethnic variation may influence root development. However, differences in CBCT acquisition protocols, voxel size, and study design should also be considered when comparing prevalence rates across studies.

### 3.3. Sex-Related Differences in Root Number

The null hypothesis assuming no association between sex and the number of roots was not rejected (*p* > 0.05). The chi-square analysis demonstrated statistically significant differences between males and females in several studies, including Bürklein et al. [41], Al-Sayaghi et al. [25], Watanabe et al. [27], Buchanan et al. [29], Pan et al. [30], de Lima et al. [33], and Bulut et al. [38] (*p* < 0.05). In contrast, no significant sex-related differences were observed in the studies by Olczak et al. [21], Erkan et al. [22], Alqedairi et al. [31], Mirza et al. [34], and Abella et al. [23] (*p* > 0.05). The greatest disparity in the number of roots was found in the studies conducted by de Lima et al. [33] (Table 1), where the prevalence of two roots was 28.4%, followed by Saber et al. [24] (Table 1) where this was 26%, and Buchanan et al. [29] (Table 1). All studies indicate a higher prevalence of two roots in men than in women.

The highest prevalence of two roots in men was found in the studies by de Lima et al. [33] (Table 4), 37.4%, Buchanan et al. [29] (Table 4), 29.7%, and Bulut et al. [38] (Table 4), 22.4%. The lowest prevalence was found in Jung et al. [28] (Table 4), 3.2%, Watanabe et al. [27] (Table 4), 5%, and Pan et al. [30] (Table 4), 12.9%.

In women, the higher prevalence of two roots was found in the studies by de Lima et al. [33] (Table 4), 23.4%, Mirza [34] (Table 4), 18%, and Abella et al. [23] (Table 4), 15.8%. The lowest prevalence was found in Jung et al. [28] (Table 4), 0.4%, Watanabe et al. [27] (Table 4), 0.8%, and Pan et al. [30] (Table 4), 4.7%. Although several studies reported statistically significant sex-related differences, these findings appear population-specific and should not be considered universal predictors of anatomical morphology. Clinical interpretation should therefore remain individualized.

### 3.4. Root Canal Configuration According to Vertucci Classification (Vc)

According to the authors, their collected research demonstrated the occurrence of all Vertucci configurations (Table 3). Compared with earlier anatomical studies based on tooth clearing techniques and conventional radiography, CBCT-based studies provide substantially improved detection of complex root canal morphology. Classical investigations by Vertucci et al. [47] and Kartal et al. [48], using dye penetration and clearing techniques, confirmed the predominance of single-rooted maxillary second premolars and simpler canal configurations. However, contemporary CBCT studies appear to report a higher prevalence of complex canal systems, particularly Vertucci types IV and V, likely because three-dimensional imaging enables more accurate visualization than traditional radiographic methods, which may be affected by anatomical superimposition. Therefore, modern CBCT imaging may be regarded not as contradictory to earlier findings, but rather as refining and expanding previous anatomical observations.

#### 3.4.1. Vertucci Type I [Vc I]

The Vertucci type I (Vc I) configuration represented the predominant canal morphology in most reviewed populations. The highest prevalence was reported in the studies conducted by Bulut et al. [38] (77.6%), Jung et al. [28] (76.5%), Watanabe et al. [27] (72.1%), and Mirza [34] (71.3%) (Table 2). In contrast, substantially lower frequencies were observed in the studies by Bürklein et al. [41] (14.3%), Saber et al. [24] (16.1%), and Elnour et al. [43] (17.0%).

#### 3.4.2. Vertucci Type II [Vc II]

The Vertucci type II (Vc II) configuration exhibited considerable variability across the reviewed populations. The highest prevalence was reported by Celikten et al. [40] (25.9%), followed by Alqedairi et al. [31] (25.8%), Lemos et al. [44] (23.9%), Abella et al. [23] (22.5%), and Saber et al. [24] (22.2%) (Table 2). In contrast, lower frequencies were observed in the studies conducted by Jung et al. [28] (5.2%), Almehrzi et al. [36] (5.3%), and Erkan et al. [22] (5.8%).

#### 3.4.3. Vertucci Type III [Vc III]

The Vertucci type III (Vc III) configuration demonstrated notable variability among the reviewed studies. The highest prevalence was reported by Almehrzi et al. [36] (32.4%), followed by Li YH et al. [42] (23.9%) and Mashyakhy et al. [32] (15.3%) (Table 2). In contrast, considerably lower frequencies were observed in the studies conducted by Bürklein et al. [41] (0.6%), Mirza [34] (0.9%), and Celikten et al. [40] (1.7%).

#### 3.4.4. Vertucci Type IV [Vc IV]

The Vertucci type IV (Vc IV) configuration was reported with considerable variability across the reviewed studies. The highest prevalence was observed in the studies by Saber et al. [24] (44.4%), Buchanan et al. [29] (33.7%), and de Lima et al. [33] (32.6%) (Table 2). In contrast, substantially lower frequencies were reported by Pan et al. [30] (1.8%), Almehrzi et al. [36] (2.5%), and Jung et al. [28] (3.1%) (Table 2).

#### 3.4.5. Vertucci Type V [Vc V]

The Vertucci type V (Vc V) configuration demonstrated considerable variability across the reviewed studies. The highest prevalence was reported by Bürklein et al. [41] (28.7%), followed by Elnour et al. [43] (23.0%) (Table 2). In contrast, markedly lower frequencies were observed in the studies by Mirza [34] (1.2%), Lemos et al. [44] (1.4%), and Erkan et al. [22], Bulut et al. [38], and Celikten et al. [40] (1.9%).

#### 3.4.6. Vertucci Type VI [Vc VI]

The Vertucci type VI (Vc VI) configuration was reported relatively infrequently across the reviewed studies. The highest prevalence was observed in the study by Bürklein et al. [41] (19.1%), followed by Watanabe et al. [27] (5.3%) and Saber et al. [24] (4.1%) (Table 2). In contrast, only minimal frequencies were reported by Bulut et al. [38] and Celikten et al. [40] (0.2%), Mirza [34] and Li YH et al. [42] (0.3%), as well as Erkan et al. [22] (0.4%). Furthermore, the studies conducted by Elnour et al. [43], Jung et al. [28], Lemos et al. [44], Ok E et al. [45], and Almehrzi et al. [36] did not report the presence of Vc VI morphology in their investigated populations (Table 2).

#### 3.4.7. Vertucci Type VII [Vc VII]

The Vertucci type VII (Vc VII) configuration was reported infrequently across the reviewed studies. The highest prevalence was observed in the study by Saber et al. [24] (7.3%), followed by Al-Sayaghi et al. [25] (2.5%) and Mashyakhy et al. [32] (2.2%) (Table 2). In contrast, lower frequencies were reported by Jung et al. [28] (0.2%), Pan et al. [30] (0.3%), and Li YH et al. [42] (0.4%). Furthermore, several studies, including those by Erkan et al. [22], Lemos et al. [44], Alqedairi et al. [31], Mirza [34], Ok E et al. [45], Bulut et al. [38], and Celikten et al. [40], did not report the presence of Vc VII morphology in their investigated populations (Table 2).

#### 3.4.8. Vertucci Type VIII [Vc VIII]

The Vertucci type VIII (Vc VIII) configuration was reported infrequently across the reviewed studies. The highest prevalence was observed in the study by Buchanan et al. [29] (2.1%), followed by Saber et al. [24] (1.2%) and Alqedairi et al. [31] (0.9%) (Table 2). In contrast, several studies, including those conducted by Abella et al. [23], Elnour et al. [43], Lemos et al. [44], Pan et al. [30], Mashyakhy et al. [32], Almehrzi et al. [36], Bulut et al. [38], Celikten et al. [40], and Li YH et al. [42], did not report the presence of Vc VIII morphology in their investigated populations (Table 2).

Taken together, the reviewed studies demonstrate that although Vertucci type I remains the predominant canal configuration in maxillary second premolars, a substantial proportion of teeth exhibit more complex anatomical patterns. Configurations corresponding to Vertucci types II–V were frequently reported and may present considerable clinical challenges due to canal bifurcations, mergers, and multiple apical exits. Rare configurations, including types VI–VIII, were identified in several populations, confirming that clinicians should not assume a simple canal anatomy during treatment planning. These findings further support the value of CBCT imaging in cases where anatomical complexity is suspected.

### 3.5. Sex-Related Differences in Vertucci Canal Configuration

Having considered all studies, the authors Olczak et al. [21], Erkan et al. [22], Bürklein et al. [41], Al-Sayaghi et al. [25], Watanabe et al. [27], Jung et al. [28], and Alqedairi et al. [31] (Table 3) took into account the gender distribution of the Vertucci configuration in their study. Table 3 shows the distribution of Vertucci configuration by gender.

The null hypothesis assumed no association between sex and Vertucci root canal configuration. The alternative hypothesis assumed that the distribution of canal configurations differs between males and females.

The chi-square test revealed statistically significant differences in the distribution of Vertucci root canal configurations between males and females in most analyzed studies (*p* < 0.05), indicating sex-related variation in canal morphology. However, in selected datasets, these differences were not statistically significant (*p* > 0.05), suggesting that the observed distributions may be population-specific rather than universally sex-dependent.

The biological mechanisms underlying sex-related differences in root canal morphology remain unclear. It has been suggested that sexual dimorphism during cranio-facial development may contribute to differences in root formation; however, the available evidence remains inconsistent. Since significant associations were observed in some populations but not in others, the clinical relevance of sex as a predictor of canal configuration should be interpreted with caution.

### 3.6. Limitations

Several limitations of the included studies must be acknowledged. Most were retrospective in design and conducted at single-center institutions, which may not fully represent the broader population.

### 3.7. Clinical Recommendation

The significant frequency of multi-canal configurations like Types Vc IV, Vc V makes the search for a second orifice mandatory in every case. Clinicians should exercise higher vigilance when treating male patients as they demonstrate a statistically higher prevalence of two rooted configurations. In complex cases, a CBCT is recommended to minimize radiation while maximizing the detection of missed canals.

#### 3.7.1. Indications for CBCT in Maxillary Second Premolars

Although CBCT offers superior visualization of root canal morphology compared with conventional periapical radiographs, its routine use for every maxillary second premolar cannot be recommended due to radiation exposure and ethical considerations. According to the ALARA and ALADA principles, CBCT should only be used when the expected diagnostic benefit outweighs radiation risk. In routine endodontic treatment, conventional periapical radiography remains the first-line imaging modality. However, CBCT should be considered in cases of suspected anatomical complexity, including inconsistent radiographic findings, sudden canal disappearance, unusual canal trajectory, retreatment cases, persistent apical pathology, or clinical suspicion of missed canals. Based on the present review, clinicians should maintain increased awareness of anatomical complexity in maxillary second premolars, particularly in populations demonstrating a higher prevalence of multi-canal and multi-rooted configurations.

#### 3.7.2. Referral to Endodontic Specialist and Treatment Complexity

The significant prevalence of complex root canal configurations, particularly Vertucci types IV and V, indicates that clinicians should maintain increased vigilance when treating maxillary second premolars. Cases involving multiple roots, abrupt canal disappearance, severe curvature, retreatment, calcification, or suspected missed anatomy may exceed the level of complexity appropriate for routine general dental practice. In such situations, referral to an endodontist or a dentist with advanced endodontic training should be considered.

#### 3.7.3. Magnification and Endodontic Equipment

Magnification tools, including dental loupes and operating microscopes, may improve the identification of additional canal orifices and anatomical irregularities. The use of enhanced illumination, ultrasonic tips, and modern irrigation systems may further increase treatment predictability in teeth with complex root canal anatomy. Furthermore, clinicians may benefit from structured case assessment systems, including the difficulty assessment tools proposed by the European Society of Endodontology (ESE) and the American Association of Endodontists (AAE), which help determine whether treatment difficulty exceeds the operator’s experience and available equipment.

## 4. Conclusions

The reviewed studies demonstrate considerable anatomical variability in the root canal configuration of maxillary second premolars. The incidence of specific root canal configurations varies among populations. An awareness of these variations is essential for accurate diagnosis and successful endodontic treatment. Further studies, particularly using advanced imaging methods, are needed to better understand the anatomical complexity of these teeth.

## Figures and Tables

**Table 1:** Distribution of the number of roots in maxillary second premolars.

Author (Ref.)	Region	Sample Size (n)	1 Root (%)	2 Roots (%)	3 Roots (%)	C-Shape (%)
Olczak et al. [21]	Poland	324	88.9	11.1	0.0	0.0
Erkan et al. [22]	Turkish-Cypriot	516	89.9	10.1	0.0	0.0
Abella et al. [23]	Spain	374	82.9	15.5	1.6	0.0
Saber et al. [24]	Egypt	342	72.8	26.0	1.2	0.0
Al-Sayaghi et al. [25]	Yemen	362	87.6	2.1	0.3	0.0
Chourasia et al. [26]	Saudi Arabia	602	78.4	20.76	0.5	0.0
Watanabe et al. [27]	Japan	717	97.8	2.0	0.3	0.0
Jung et al. [28]	Korea	578	97.6	1.9	0.5	0.0
Buchanan et al. [29]	South Africa	285	78.2	20.4	1.4	0.0
Pan et al. [30]	Malaysia	333	91.9	8.1	0.0	0.0
Alqedairi et al. [31]	Saudi Arabia	318	85.2	14.5	0.3	0.0
Mashyakhy et al. [32]	Saudi Arabia	359	88.0	12.0	0.0	0.0
de Lima et al. [33]	Brazil	503	71.2	28.4	0.4	0.0
Mirza et al. [34]	Saudi Arabia	680	83.08	16.47	0.45	0.0
Mustafa et al. [35]	Saudi Arabia	303	99.7	0.3	0.0	0.0
Almehrzi et al. [36]	UAE	521	91.0	7.8	0.6	0.6
Algarni et al. [37]	Pakistan	1047	93.2	6.8	0.0	0.0
Bulut et al. [38]	Turkey	476	82.1	17.9	0.0	0.0
Estrela et al. [39]	Brazil	100	83.0	17.0	0.0	0.0
Celikten et al. [40]	Turkish-Cypriot	479	85.4	14.2	0.4	0.0
Bürklein et al. [41]	Germany	512	82.8	17.0	0.4	0.0
Li YH et al. [42]	China	1403	96.2	3.8	0.0	0.0

**Table 2:** Distribution of Vertucci root canal configurations in maxillary second premolars.

Author (Ref.)	Sample Size (n)	Vc I (%)	Vc II (%)	Vc III (%)	Vc IV (%)	Vc V (%)	Vc VI (%)	Vc VII (%)	Vc VIII (%)	Non-Vertucci (%)
Olczak et al. [21]	324	59.6	9.3	6.2	15.7	7.1	0.9	0.9	0.3	0.0
Erkan et al. [22]	516	57.4	5.8	5.4	28.9	1.9	0.4	0.0	0.2	0.0
Abella et al. [23]	374	39.3	22.5	7.2	19.8	4.3	3.2	2.1	0.0	0.0
Bürklein et al. [41]	512	14.3	11.1	0.6	25.0	28.7	19.1	0.6	0.6	0.0
Saber et al. [24]	342	16.1	22.2	1.8	44.4	2.9	4.1	7.3	1.2	0.0
Al-Sayaghi et al. [25]	362	46.1	9.9	14.1	13.2	9.3	3.0	2.5	0.3	0.0
Elnour et al. [43]	100	17.0	7.0	9.0	23.0	23.0	0.0	2.0	0.0	0.0
Watanabe et al. [27]	717	72.1	8.9	5.3	9.5	3.5	5.3	0.5	0.3	0.0
Jung et al. [28]	578	76.5	5.2	9.7	3.1	4.8	0.0	0.2	0.5	0.0
Buchanan et al. [29]	285	37.5	11.9	5.3	33.7	7.4	1.4	0.7	2.1	0.0
Lemos et al. [44]	284	47.2	23.9	2.8	24.7	1.4	0.0	0.0	0.0	0.0
Pan et al. [30]	333	61.3	18.0	9.3	1.8	6.3	3.0	0.3	0.0	0.0
Alqedairi et al. [31]	318	49.4	25.8	5.0	11.6	5.7	1.6	0.0	0.9	0.0
Mashyakhy et al. [32]	359	38.2	10.9	15.3	19.2	12.3	1.1	2.2	0.0	0.8
de Lima et al. [33]	503	49.9	9.3	2.2	32.6	4.0	0.8	0.8	0.4	0.0
Mirza et al. [34]	680	71.3	7.4	0.9	18.2	1.2	0.3	0.0	0.7	0.0
Ok E et al. [45]	1301	54.5	8.84	3.61	21.91	10.84	0.0	0.0	0.3	0.0
Almehrzi et al. [36]	521	47.4	5.3	32.4	2.5	10.9	0.0	0.9	0.0	0.6
Bulut et al. [38]	476	77.6	12.5	1.3	6.5	1.9	0.2	0.0	0.0	0.0
Celikten et al. [40]	479	45.9	25.9	1.7	16.9	1.9	0.2	0.0	0.0	0.0
Li YH et al. [42]	1403	50.3	10.4	23.9	5.9	8.0	0.3	0.4	0.0	0.7

**Table 3:** Distribution of Vertucci root canal configurations in maxillary second premolars by sex with chi-square comparison.

Author	Male n	Vc I %	Vc II %	Vc III %	Vc IV %	Vc V %	Vc VI %	Vc VII %	Vc VIII %	Female n %	Vc I %	Vc II %	Vc III %	Vc IV %	Vc V %	Vc VI %	Vc VII %	Vc VIII %	χ^2^	*p*-Value
Olczak et al. [21]	136	54.4	8.1	5.1	19.9	10.3	2.2	0.0	0.0	188	63.3	10.1	6.9	12.8	4.8	0.0	1.6	0.5	14.677	**0.0404**
Erkan et al. [22]	217	64.7	4.5	2.3	23.9	3.2	0.2	0.0	1.1	299	68.0	2.9	1.9	23.9	2.8	0.3	0.0	0.3	2.585	0.8588
Bürklein et al. [41]	235	21.0	5.5	0.5	18.8	51.1	2.8	0.3	0.5	277	22.6	5.2	0.0	11.7	59.1	0.9	0.5	0.0	11.4	0.1221
Al-Sayaghi et al. [25]	180	39.4	8.9	12.2	18.3	10.6	6.1	3.3	0.0	182	52.7	11.0	15.9	8.2	8.2	0.0	1.6	0.5	25.359	**<0.001**
Watanabe et al. [27]	201	68.2	10.0	7.0	13.9	1.0	0.0	0.0	0.0	516	73.6	8.5	4.7	7.7	4.5	0.4	0.2	0.4	15.312	**0.0322**
Jung et al. [28]	311	74.0	6.1	8.7	2.6	7.7	0.0	0.0	1.0	267	79.4	4.1	10.9	3.7	1.5	0.0	0.4	0.0	18.267	**0.0056**
Alqedairi et al. [31]	159	48.1	27.5	3.8	13.8	6.2	0.0	0.0	0.6	158	50.6	24.0	6.3	9.5	5.1	3.2	0.0	1.3	8.383	0.2114

Note: Statistically significant differences (*p* < 0.05) are shown in bold.

**Table 4:** Distribution of the number of roots in maxillary second premolars by sex with chi-square comparison.

Author	Male n	1 Root (%)	2 Roots (%)	3 Roots (%)	Female n	1 Root (%)	2 Roots (%)	3 Roots (%)	χ^2^	*p*-Value
Olczak et al. [21]	136	84.6	15.4	0.0	188	92.0	8.0	0.0	3.66	0.0557
Erkan et al. [22]	217	83.2	16.8	0.0	299	88.2	11.8	0.0	2.225	0.1358
Bürklein et al. [41]	235	78.7	20.5	0.9	277	86.3	14.1	0.0	6.437	**0.0400**
Al-Sayaghi et al. [25]	180	82.8	17.2	0.0	182	92.3	7.1	0.5	9.448	**0.0089**
Watanabe et al. [27]	101	95.0	5.0	0.0	516	98.8	0.8	0.2	10.329	**0.0057**
Jung et al. [28]	311	95.8	3.2	1.0	267	99.6	0.4	0.0	8.79	**0.0123**
Buchanan et al. [29]	128	67.2	29.7	3.1	157	87.3	12.7	0.0	18.528	**<0.001**
Pan et al. [30]	146	87.1	12.9	0.0	193	95.3	4.7	0.0	6.353	**0.0117**
Alqedairi et al. [31]	159	83.1	16.3	0.6	158	87.3	12.7	0.0	1.82	0.4025
de Lima et al. [33]	182	62.6	37.4	0.0	321	76.0	23.4	0.6	12.02	**0.0025**
Mirza et al. [34]	279	83.1	15.8	1.1	401	81.5	18.0	0.5	1.311	0.5192
Bulut et al. [38]	237	77.6	22.4	0.0	239	86.6	13.4	0.0	5.966	**0.0146**
Abella et al. [23]	204	83.3	15.2	1.5	170	82.4	15.8	1.8	0.082	0.9600

Note: Statistically significant differences (*p* < 0.05) are shown in bold.

## Data Availability

No new data were created or analyzed in this study. Data sharing is not applicable to this article.
